# Limb-shaking TIA: a case of cerebral hypoperfusion in severe cerebrovascular disease in a young adult

**DOI:** 10.1186/s12883-021-02296-4

**Published:** 2021-07-03

**Authors:** Tom E. Richardson, Paul Beech, Geoffrey C. Cloud

**Affiliations:** 1 Department of Neurology, Alfred Hospital, Melbourne, Australia; 2 Department of Radiology, Alfred Hospital, Melbourne, Australia; 3 Department of Clinical Neuroscience, Central Clinical School, Monash University, Melbourne, Australia

**Keywords:** Stroke, Cerebrovascular disease, Limb-shaking TIA, Cerebral perfusion imaging

## Abstract

**Background:**

Limb-shaking transient ischaemic attacks (TIAs) are an under recognised presentation of severe cerebrovascular disease resulting from cerebral hypoperfusion. Patients present with jerking, transitory limb movements precipitated by change in position or exercise that are often confused with seizure. Cerebral perfusion imaging studies are an important tool available to aid diagnosis.

**Case presentation:**

We present the case of a young female who developed limb-shaking TIA in the context of progressive severe intracranial atherosclerotic disease (ICAD). Previous cortical infarction raised suspicion for seizure as a cause of her symptoms. However, single photon emission CT (SPECT) with CT acetazolamide challenge identified severe left hemisphere cerebral hypoperfusion and a diagnosis of limb-shaking TIA was made. Symptoms improved with maximal medical management.

**Conclusions:**

This case highlights the importance of cerebral perfusion imaging for diagnostic confirmation as well as therapeutic options available to alleviate symptoms and reduce stroke risk in patients with limb-shaking TIA.

## Background

Historically limb-shaking transient ischaemic attacks (TIA) has been thought as a rare neurological entity, yet has been identified in up to 29% of patients with symptomatic internal carotid artery (ICA) occlusion at presentation [1]. Conservative estimates suggest an incidence for symptomatic ICA occlusion of 6 per 100,000 persons, responsible for 15% of all large vessel infarctions [2]. The presence of limb-shaking TIA in these patients doubles the risk for poor clinical outcomes in the short and long term, as measured by the modified Rankin Scale, and increases the risk of recurrent stroke and TIA [1]. This suggests an under recognition of the prevalence and importance of limb-shaking TIA.

We present a case of limb-shaking TIA in a patient with extensive intracranial atherosclerotic disease (ICAD), the utility of cerebral perfusion imaging studies to differentiate from seizure, and the therapeutic options available for these patients.

## Case presentation

A 34-year-old female with recurrent ischaemic stroke, type 1 diabetes mellitus, hypertension, dyslipidaemia and stage 2 chronic kidney disease, presented to the outpatient stroke clinic in June 2020 with a history of recurrent transitory right leg weakness and right hand shaking. These episodes occurred several times per day lasting approximately 30 s whilst mobilising and resolved on sitting. Neurological examination demonstrated recovering dysphasia and mild pyramidal right-sided weakness requiring a walking stick for gait assistance.

**Fig. 1:**
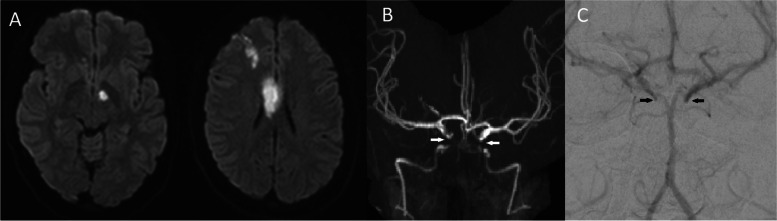
**a** MR of brain (diffusion weighted image) demonstrating multiterritory acute infarct; **b** Time of flight MR demonstrating bilateral ICA occlusion; and **c** DSA demonstrating anterior circulation supplied by posterior circulation

Previously, in March 2019, she presented with slurred speech, left-sided weakness and visual neglect with confirmed bilateral embolic middle cerebral artery (MCA) territory infarcts on magnetic resonance imaging (MRI). Computer tomography (CT) carotid angiography and Digital subtraction angiogram (DSA) identified severe ICAD with bilateral occlusion of the supraclinoid segment of the internal carotid arteries but no features of moyamoya disease (Fig. 1). She had no family history of stroke, a negative thrombophilia screen and no cardioembolic source identified. Secondary prevention was commenced including aggressive lipid lowering with rosuvastatin aiming for low density lipoprotein < 1.8 mmol/L, dual antiplatelet therapy with aspirin and clopidogrel for three months, antihypertensive treatment aiming for blood pressure < 130/80 mmHg and augmentation of her diabetic regime. She recovered to a point of supported independent living but was unable to return to work.

In March 2020 she represented to the emergency department with reduced consciousness in a state of diabetic ketoacidosis secondary to a lower respiratory tract infection and episodes of vomiting. On presentation her blood pressure was 70/40 mmHg and she was found to have new right-sided weakness. Diffusion weighted MRI identified multiple acute embolic infarcts in the left MCA territory. Her deficits improved with a period of neurorehabilitation but she had ongoing high-level language and cognitive difficulties, marked post stroke fatigue as well as reduced high-level balance. She was independent in personal care.

**Fig. 2:**
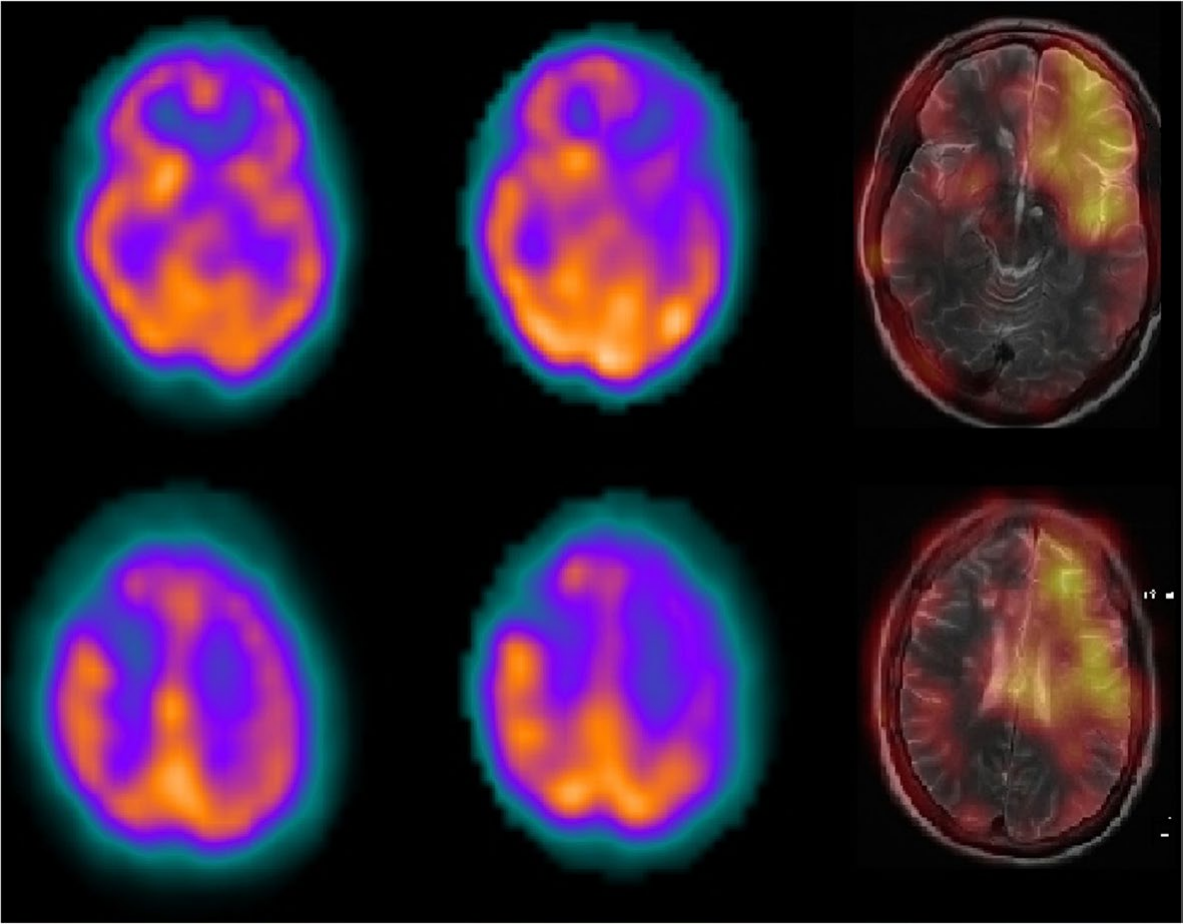
Two axial images of 99mTc-ethyl cysteine dimer cerebral perfusion SPECT/CT at rest (left) with acetazolamide challenge (middle) and a subtracted image of resting perfusion minus acetazolamide challenge with fusion to the T2 weighted MRI sequences (right) demonstrating the area of acetazolamide induced hypoperfusion

**Fig. 3:**
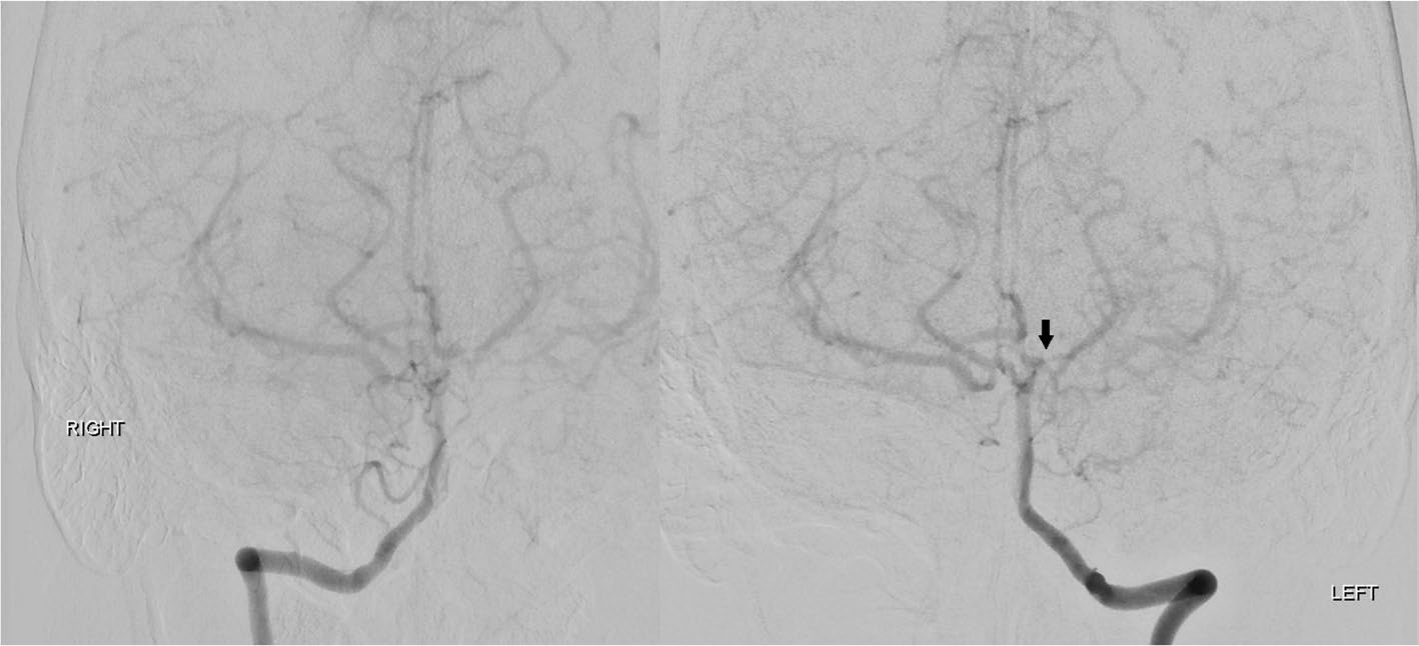
DSA with bilateral vertebral artery injections demonstrating worsening perfusion of the left anterior circulation with progressive stenosis of the left posterior communicating artery

In neurorehabilitation initial concerns were for post stroke focal motor seizures causing her transient symptoms and she was commenced on levetiracetam. However, these brief episodes spared the face, did not exhibit Jacksonian march and were precipitated by periods of reduced cerebral perfusion such as changing position and dehydration. When reviewed in stroke clinic the leading differential diagnosis was cerebral hypoperfusion syndrome. A 99mTc-ethyl cysteine dimer cerebral perfusion single photon emission CT (SPECT) with CT acetazolamide challenge was performed (Fig. 2). Baseline perfusion was reduced in the left frontal, temporal and parietal lobes in keeping with recent infarction as well as a small fixed perfusion defect in the right frontal lobe corresponding to previous established infarct. Although asymptomatic during scanning, worsening hypoperfusion to the left frontal lobe and to a lesser extent the left temporal and anterior parietal lobes, was evident with acetazolamide challenge, indicative of reduced vascular reserve in these territories. Given her ongoing symptoms and declining quality of life a repeat DSA was performed to assess for possible sites of surgical revascularisation. The second DSA demonstrated progressive intracranial stenosis with opacification of the left posterior communicating artery and collateral supply to the left hemisphere predominantly from the posterior choroidal artery and posterior cerebral artery (Fig. 3). No feasible site for surgical revascularisation was identified. She was managed on maximal medical therapy including long term dual antiplatelet therapy, consolidated antihypertensive regime, tight glycaemic control and avoidance of dehydration. Over the following six months a systolic blood pressure between 120 and 130/70 mmHg was achieved, with a low-density lipoprotein of 1.0 mmol/L and high-density lipoprotein of 1.2 mmol/L. Glycaemic control remained an ongoing challenge, with a glycosylated haemoglobin level of 9.5% (80 mmol/mol). The patient has recently trialled a continuous infusion pump device. Levetiracetam was ceased. During this period the limb-shaking TIAs settled.

## Discussion and conclusion

Limb-shaking TIAs are a neurological phenomenon characterised by rhythmic, jerking choreatic like movements affecting the upper and lower limb. They are a form of haemodynamic TIA, occurring in patients with high grade stenoses or occlusion of the ICAs or MCAs [3]. Patients are reliant on collateral supply to ensure cerebral perfusion. During periods of changing haemodynamics, collateral supply may be insufficient to maintain adequate cerebral perfusion, likely leading to hypoperfusion induced transient ischaemia in watershed territories [4, 5]. As seen in our case, symptom onset is usually precipitated by standing, mobilising or exercising and resolves quickly with activity cessation.

Limb-shaking TIA may often be confused with seizure, an important differential diagnosis that must be excluded. As seizures are a common complication post stroke [6], past history cannot always guide the diagnosis. With our case, there was initial concerns for seizure given her past history of recurrent MCA strokes with cortical involvement in the left hemisphere within twelve months, potentially creating an epileptogenic focus. Key features of seizures do not occur in limb-shaking TIA and electroencephalography does not show epileptiform activity (Table 1) [5].

**Table 1:** Features of limb-shaking TIA compared to seizure

	**Limb-shaking TIA**	**Seizure**
Symptoms	Rhythmic jerking Transient paraesthesia Face sparing	Altered consciousness Motor (automatisms, tonic, clonic, myoclonic, spasms, hyperkinesis) Nonmotor (autonomic, cognitive, emotional, sensory)
Duration	< 5 min	< 30 min
Precipitants	Exercise Changing position Hypovolaemia Antihypertensives	Hyperventilation Intercurrent infection/sepsis Altered alcohol intake Antiepileptic noncompliance

Ongoing suspicion for limb-shaking TIA requires vascular imaging to establish the presence of cerebrovascular disease. CT or MR angiography, Duplex ultrasound and transcranial doppler ultrasound are commonly used, however catheter angiography remains the gold standard [7]. Diffusion weighted MR and CT studies may show areas of infarction in border-zone regions suggestive of watershed infarct [8]. In our case, the presence of bilateral ICA occlusion and progressive ICAD on DSA, raised suspicion for cerebral hypoperfusion causing limb-shaking symptoms.

Assessment of cerebral hypoperfusion requires dynamic cerebral perfusion studies with acetazolamide or C0_2_ challenge. Multiple neuroimaging modalities are available, including cerebral perfusion SPECT/CT, CT perfusion, arterial spin labelling or contrast enhanced MR perfusion and positron emission tomography (PET), each with its own advantages and disadvantages (Table 2). There is currently no recognised gold standard for assessing cerebral hypoperfusion and vascular reserve.

**Table 2:** Considerations for imaging modality to assess for cerebral hypoperfusion and cerebrovascular reserve

	**Advantages**	**Disadvantages**
SPECT/CT	Can be performed in supine and standing position to assess for orthostatic changes in cerebral perfusion Radiotracer can be administered during clinical events to differentiate hypoperfusion events with epileptic hyperperfused events	Greater effective dose of whole body radiation in comparison to MRI, PET or CT perfusion
CT Perfusion	Fast scan times	High target organ radiation to the head Nephrotoxic effects of iodine based contrast
Arterial Spin Labelling MRI	No radiation No contrast required	Low signal to noise ratio requiring prolonged scan times (high risk of movement artefact) Patient intolerance to MRI (claustrophobia, large body habitus)
Contrast enhanced perfusion MRI	No radiation	Patient intolerance to MRI (claustrophobia, large body habitus) Gadolinium contrast risks
PET	Gold standard cerebral blood flow quantitation	Short lived radiotracer requires on-site cyclotron Technically demanding

SPECT/CT allows for an accurate anatomical assessment of cerebral function and perfusion and its utility in assessing limb-shaking TIA has been demonstrated in several similar cases [9, 10]. Imaging at the time of an event can demonstrate hyperperfusion, suggestive of seizure, and identify epileptogenic foci [11]. Combining SPECT/CT with acetazolamide challenge provides valuable information about physiologically significant vascular flow reserve. In our case, worsening hypoperfusion in the left hemisphere with acetazolamide challenge suggests likely occurrence of acetazolamide steal syndrome and reduced vascular reserve capacity. Avoiding kidney injury also favoured the use of SPECT/CT.

However, a key disadvantage of SPECT/CT is radiation exposure. The use of CT imaging and radio-labelled isotopes exposes patients to external x-ray and internal gamma-ray radiation respectively. Although ionising x-ray radiation to the brain is lower with SPECT/CT compared to other CT modalities, such as CT perfusion, the use of a radionuclide results in a greater total whole-body radiation dose [12, 13]. Given the natural history of disease progression, and therefore the potential need for repeat imaging, the cumulative radiation dose with SPECT/CT is an important consideration. Arterial spin labelling MRI is a radiation-free alternative that may be more appropriate for longitudinal monitoring, however practical issues around availability and movement artefact are an ongoing barrier to use [14].

Management principles of limb-shaking TIA focus on restoring cerebral blood flow, preventing atherosclerotic disease progression and reducing stroke risk. Therapeutic approaches depend on the location of cerebrovascular disease and chronicity of presentation. Carotid endarterectomy (CEA) is the treatment of choice for reducing stroke risk and alleviating limb-shaking TIA complicating high-grade extracranial ICA stenosis, although in patients under 70 years old, carotid stenting with an experienced proceduralist may be reasonable [10, 15–18]. If limb shaking TIA complicates acute extracranial ICA occlusion it is usually short lived whilst collateralisation via the circle of Willis compensates. These patients benefit from avoiding hypovolaemia and hypotension as well as bed rest in the short term, however randomised controlled trials are lacking [19]. For patients with chronic ICA occlusion who develop limb-shaking TIA, practice at our centre is to cautiously taper antihypertensives to alleviate symptoms [20]. Unlike in high-grade stenosis, surgical revascularisation of extracranial ICA occlusion is controversial. Both CEA and extracranial to intracranial (EC-IC) bypass have not been shown to reduce long-term stroke risk in ICA occlusion [21, 22]. In these patients vascular reserve capacity is an important parameter for successful revascularisation, with poorer outcomes associated with reduced vascular reserve on dynamic perfusion imaging [23].

For patients with ICAD the influential SAMMPRIS trial resulted in aggressive medical management with aspirin, clopidogrel, lipid lowering therapy and blood pressure control combined with walking exercise becoming the mainstay over endovascular stenting for secondary stroke prevention [24, 25]. However, the prevalence of limb-shaking TIA in the SAMMPRIS cohort is not known. One case study of limb-shaking TIA in the context of M1 MCA occlusion has demonstrated resolution of symptoms with the use of a wingspan stent [9]. Superficial temporal artery to MCA bypass is another option in ICAD, which has been shown to improve haemodynamics and reduce stroke risk in highly selected cases [26]. In our case, the extent of intracranial stenosis precluded surgical revascularisation and overtime symptom improvement was achieved with maximal medical therapy and likely ongoing collateralisation.

In conclusion limb-shaking TIA is an important clinical manifestation of cerebral hypoperfusion in severe cerebrovascular disease. Early recognition and assessment with neuroimaging, including perfusion studies, are important for confirming the diagnosis and guiding management.

## Data Availability

All data and material supporting this case are contained within the manuscript.

## Abbreviations

TIA: Transient ischaemic attacks
ICA: Internal carotid artery
ICAD: Intracranial atherosclerotic disease
MCA: Middle cerebral artery
MRI: Magnetic resonance imaging
CT: Computer tomography
DSA: Digital subtraction angiography
SPECT: Single photon emission CT
PET: Positron emission tomography
CEA: Carotid endarterectomy
EC-IC: Extracranial to intracranial
